# Identification of volatile active components in Acori Tatarinowii Rhizome essential oil from different regions in China by C6 glioma cells

**DOI:** 10.1186/s12906-020-03020-4

**Published:** 2020-08-17

**Authors:** Lu Yan, Zhanzhan Liu, Li Xu, Yiyun Qian, Pingping Song, Min Wei

**Affiliations:** 1 Institute of Botany, Jiangsu Province and Chinese Academy of Sciences, Nanjing, 210014 China; 2 Jiangsu Key Laboratory for the Research and Utilization of Plant Resources, Nanjing, 210014 China; 3 The Jiangsu Provincial Platform for Conservation and Utilization of Agricultural Gerplasm, Nanjing, 210014 China; 4 North Information Control Research Academy Group Co., Ltd., Nanjing, 211153 China; 5 State Key Laboratory of Environmental Chemistry and Ecotoxicology, Research Center for Eco-Environmental Science, Chinese Academy Sciences, Beijing, 100085 China

**Keywords:** Acori Tatarinowii Rhizome, Essential oil, Region difference, Volatile active component, Neuroprotective

## Abstract

**Background:**

Acori Tatarinowii Rhizome (ATR) is a well-recognized Chinese herbal medicine prescribed to treat neurological disorders. The essential oil (ATEO) is considered as the active fraction of ATR and the content of ATEO is used as the only indicator for ATR content determination. The quality of ATEO varies widely due to region difference; however, little is known about how to study ATEO quality chemically and biologically in response to region difference. Thus, it is of great importance to identify volatile active components in ATEO to conduct quality study. In this study, we analyzed ATEO from different regions in China using chemical component analysis combined with biological activity evaluation.

**Methods:**

GC-MS was used to obtain different volatile component profiles of ATEO and significantly changed volatile components were screened out. The neuroprotective activities of ATEO, including anti-oxidation, anti-inflammation and neurotrophic functions, were revealed in C6 glioma cells. The correlation study between the bioactivities and the components was performed.

**Results:**

57 volatile components, including terpenoids, phenylpropanoids, aromatic compounds, and other aliphatic compounds, were identified. 8 volatile components (β-asarone, cis-methyl isoeugenol, γ-asarone, methyleugenol, calarene, longifolene, β-caryophyllene and caryophyllene oxide) from ATEO were significantly changed due to region difference and 2 of them (β-asarone and γ-asarone) showed strong correlation with neuroprotective activities.

**Conclusions:**

Our results reveal that ATEO from different regions in China show great changes in chemical composition and biological activity. Moreover, phenylpropanoids (β-asarone and γ-asarone) present strong correlation with the bioactivities, which are considered as volatile active components in ATEO. The findings will be useful for the development of quality study of ATEO.

## Background

The rigorous implementation of Drug Administration Law (DAL) and Good Agricultural Practices (GAP) plays a key role in promoting the quality study of Chinese herbal medicine and ensuring the safety, efficacy, and quality consistency of final drugs [1]. Chinese herbal raw materials from diverse regions undergo standard operating procedures during planting, processing and pharmaceutical production. Thus, the region difference is likely to be one of the major reasons for the heterogeneous quality of final drugs as the difference directly affects chemical composition and biological activity of Chinese herbal medicine [2, 3]. However, current quality study approaches for Chinese herbal medicine are not able to perform an overall evaluation on region difference [4]. Therefore, to achieve an improvement in quality study, the impact of region should be under serious consideration. Appropriate research is required to deepen the knowledge of quality study with region difference; this knowledge will enhance the regulation and quality of Chinese herbal medicine [5, 6].

Acori Tatarinowii Rhizoma (ATR, the dried rhizome of *Acorus tatarinowii* Schott) is one of the most important Chinese herbal medicines, which has been used in treating neurological disorders for thousands of years [7]. Extracts of ATR enhance neurogenesis and neuroprotection in both animal and clinical studies [8–10]. According to Chinese Pharmacopoeia, the major active fraction of ATR is the essential oil, and the content of ATR essential oil (ATEO) is considered as the only indicator for ATR content determination [11]. ATEO is reported to contain different groups of chemical constituents including terpenoids, phenylpropanoids and aromatic compounds, which have neuroprotective activities as anti-oxidation, anti-inflammation and neurotrophic function [12–15].

Our study on resources of Chinese herbal medicine has found that *Acorus tatarinowii* Schott shows advantage of wide growth adaptability which is mainly distributed to the south of the Yellow River in China. Six major regions in China include mountain areas in Anhui (AH), Hubei (HB) and Yunnan (YN) provinces, valley area in Hunan (HN) province, hilly area in Jiangxi (JX) province and hilly basin area in Zhejiang (ZJ) province. These regions present great differences in geographical environment, and it seems likely that the diversity leads to various problems, including an uneven quality of ATEO and its products [16]. However, there are few reports to compare ATEO quality in these regions, and greater efforts are needed to study ATEO quality with region diversity.

Region difference causes variation in chemical composition and consequently affects bioactivity of herbal medicine, and determining how to chemically and biologically analyze samples from different regions is the key to study the quality of ATEO [17, 18]. First, the variation in chemical composition may reflect the changes in type and amount of components. However, few studies clearly illustrate the variation of chemical composition in ATEO from different regions. Second, changes in type and amount of components in response to region difference would lead to changes in biological activity of ATEO. The beneficial effects of ATEO may not be attributed to one single substance, but are believed to involve a series of active components found in ATEO [19]. Study on biological activity analyzes the effects of all the active components in ATEO and the action of each component. Therefore, finding volatile active components in ATEO from different regions in China using chemical component analysis combined with biological activity evaluation is applied to explore quality study with region difference.

In the study, we aimed to identify volatile active components in ATEO from six major regions in China. GC-MS was used to obtain different volatile component profiles of ATEO and significantly changed volatile components were identified by one-way ANOVA and partial least-squares discriminant analysis (PLS-DA). Astrocytes are the major glial cells in the central nervous system and play a crucial role in neuroprotection, including anti-oxidation, anti-inflammation and neurotrophic function [20–22]. Thus, C6 glioma cells, an astrocyte-like cell line, were selected as the cell model to investigate the neuroprotective activities of ATEO samples [23]. We then analyzed the correlation between the bioactivities and the components to figure out volatile active components in ATEO. Our results reveal that ATEO samples from six major regions in China show great changes in chemical composition and biological activity. Moreover, phenylpropanoids (β-asarone and γ-asarone) present strong correlation with the bioactivities, which are considered as volatile active components in ATEO. The findings will be useful for the development of quality study of ATEO, which, in turn, enhance the medicinal benefits and find good resources of ATR.

## Methods

### Reagents and chemicals

n-Alkanes were purchased from Shanghai ANPEL Scientific Instrument (Shanghai, China). Ultra-pure water was prepared from a Milli-Q purification system (Millipore, Molsheim, France). N-hexane and other reagents were from Sigma-Aldrich (St. Louis, MO).

### Preparation of ATEO

ATR samples from six major regions were obtained from Bozhou Market in Anhui China, and were morphologically authenticated by Dr. Min Wei, Institute of Botany, Jiangsu Province and Chinese Academy of Sciences, Nanjing. The sampling details are shown in Table 1 and Additional file 1. The corresponding voucher specimens were deposited in Research Center of Medicinal Plants of Institute of Botany, Jiangsu Province and Chinese Academy of Science. The plant materials were tested for quality according to the requirements of Chinese Pharmacopeia (2015 Edition). In preparing the essential oil, 100 g of the plant materials were minced and soaked in 800 mL of water for 1 h. The mixture was submitted to hydro-distillation in a volatile oil extractor for 8 h [11, 13]. The resultant ATEO was collected, dried over anhydrous sodium sulfate and stored at − 20 °C.

**Table 1:** Details of ATEO samples from six major regions used in this study

ATEO samples	Locality
ATEO-AH	Yuexi Country, Anqing, Anhui (AH)
ATEO-HB	Yidu Country, Yichang, Hubei (HB)
ATEO-HN	Yuanling Country, Huaihua, Hunan (HN)
ATEO-JX	Taihe Country, Ji’an, Jiangxi (JX)
ATEO-YN	Tengchong Country, Baoshan, Yunnan (YN)
ATEO-ZJ	Pan’an Country, Jinhua, Zhejiang (ZJ)

### GC-MS analysis

GC-MS analysis was performed on a TRACE 1300/ISQ-LT (Thermo Fisher Scientific, Waltham, MA). The chromatographic separation was conducted on an Agilent J&W DB-5 capillary column (30 m × 0.25 mm, 0.25 μm, Agilent Technologies, Santa Clara, CA) using the following temperature profile: 60 °C for 4 min, 60–150 °C at 12 °C/min, 150–170 °C at 1 °C/min, 170–250 °C at 30 °C/min, and held for 3 min; inlet temperature, 250 °C; transfer-line temperature, 250 °C; split ratio, 1:33.3. The mass spectrometer was operated in the electron impact (EI) mode with energy of 70 eV, and data were collected at a rate of 5 scan/s over a range of m/z 33–450. The ion source was kept at 250 °C [24]. For qualitative analysis of ATEO, the individual peak was identified by comparing their mass spectra with the mass spectral library (NIST14), with MS spectra and MS fragmentation pattern published in the literature and with relative retention index by injecting a mixture of n-alkanes. For relative quantification of ATEO, the individual peak was integrated manually and their relative contents were calculated by peak area normalization method.

### Cell culture

C6 glioma cells (ATCC® CCL-107) were obtained from American Type Culture Collection (ATCC, Manassas, VA), and were cultured in Dulbecco’s modified Eagle’s medium (DMEM), supplemented with 10% fetal bovine serum (FBS), 100 units/mL penicillin and 100 μg/mL streptomycin in a humidified CO_2_ (5%) incubator at 37 °C. Culture medium was changed every other day. All culture reagents were purchased from Thermo Fisher Scientific.

### Drug treatment

In brief, cultured C6 cells were treated with different concentrations of ATEO (1.5, 5, 15 μg/mL) for 24 or 48 h. The culture medium was replaced 3 h prior to drug treatment. In luciferase assay, the cells were pre-treated with ATEO (1.5, 5, 15 μg/mL) for 24 h. Forskolin (FSK, Sigma, St. Louis, MO, 10 μM) and ZLN005 (MCE, Monmouth Junction, NJ, 2 μM) were used as positive control, respectively. In mRNA assay, cultured C6 cells were treated with ATEO (15 μg/mL) for 48 h with or without tumor necrosis factor-α (TNF-α, Sigma, St. Louis, MO, 10 ng/mL) and interferon-γ (IFN-γ, Sigma, St. Louis, MO, 10 ng/mL) pretreatment for another 24 h [25].

### Luciferase assay

Two promoter constructs were purchased from FulenGen (Guangzhou, China), namely pCRE-Luc and pPGC-1α-Luc, carrying cAMP response element (CRE) and peroxisome proliferator-activated receptor-γ coactivator-1α (PGC-1α) promoter sequences, respectively. Cultured C6 cells were seeded in 12-well plates at a density of 1 × 10^5^ per well and stayed overnight. Then the cells were transiently transfected with pCRE-Luc and pPGC-1α-Luc by lipofectamine 3000 reagent (Thermo Fisher Scientific), respectively [26]. After drug treatment, the cells were harvested and luciferase activity was measured using the Luc-Pair™Duo-Luciferase HS Assay kit (GeneCopoeia, Rockville, MD). Firefly luciferase activity was normalized to Renilla luciferase activity.

### Real-time quantitative PCR

Total RNA was isolated from cell cultures by RNA Isolation Kit (Vazyme, Nanjing, China) according to the manufacturer’s instructions. The concentrations of RNAs were detected by UV absorbance at 260 nm. cDNA was reverse transcribed from 1 μg samples of total RNA using RT SuperMix for qPCR (Vazyme), according to the protocol provided by the manufacturer. Real-time PCR was performed using SYBR Green Master Mix (Vazyme). The SYBR green signal was detected by qTOWER 2.0 (Analytic Jena AG, Germany). Primers used were: 18S-S: TGT GAT GCC CTT AGA TGT CC; 18S-AS: GAT AGT CAA GTT CGA CCG TC; NGF-S: CAC TCT GAG GTG CAT AGC GTA ATG TC; NGF-AS: CTG TGA GTC CTG TTG AAG GAG ATT GTA C; BDNF-S: GAG CTG AGC GTG TGT GAC AGT ATT AG; BDNF-AS: ATT GGG TAG TTC GGC ATT GCG AGT TC; GDNF-S: GCG CTG ACC AGT GAC TCC AAT ATG; GDNF-AS: CGC TTC ACA GGA ACC GCT ACA ATA TC; GPx1-S: GGA CTA CAC CGA AAT GAA TGA TCT G; GPx1-AS: GAA GGT AAA GAG CGG GTG AGC; SOD2-S: GCC AAG GGA GAT GTT ACA ACT CAG; SOD2-AS: GCA GTG GGT CCT GAT TAG AGC AG; UCP2-S: ACG ACC TCC CTT GCC ACT TCA C; UCP2-AS: CAA GCG GAG GAA GGA AGG CAT G; IL-1β-S: GAT GAA AGA CGG CAC ACC CAC C; IL-1β-AS: GAG AGG TGC TGA TGT ACC AGT TG; IL-6-S: CTG GAG TTC CGT TTC TAC CTG GAG; IL-6-AS: GAT GGT CTT GGT CCT TAG CCA CTC; TNF-α-S: GAC CCT CAC ACT CAG ATC ATC TTC; TNF-α-AS: GTG GGT GAG GAG CAC ATA GTC G.

### Statistical analysis

The resulting GC-MS datasets were analyzed using an online tool, MetaboAnalyst 4.0 (www.metaboanalyst.ca) [27]. Data were normalized by sum and auto scaling. One-way ANOVA, clustering analysis and PLS-DA were used for classification analysis. In PLS-DA, the quality of the fitting model can be explained by the appropriate R^2^ and Q^2^ values. R^2^ is defined as the total amount of variation explained by the model and Q^2^ is the indicated predictability of the model under cross validation. The PLS-DA was validated by a permutation tests (1000 random iterations) [28]. A multi-criteria assessment (MCA), including variable importance in projection (VIP) values and *p* values, were used to screen and select significantly changed volatile components. The MCA was performed using the followed criteria: 1. VIP > 1 and 2. *p* < 0.05 [29]. The correlation analysis between activity data and relative contents was performed using RStudio 1.1.463 for R statistical computing (www.rstudio.com/).

All data were analyzed using one-way ANOVA or Students t-test method. Differences with values of *p* < 0.05 were considered significant.

## Results

### Different volatile component profiles of ATEO from six major regions in China

**Fig. 1:**
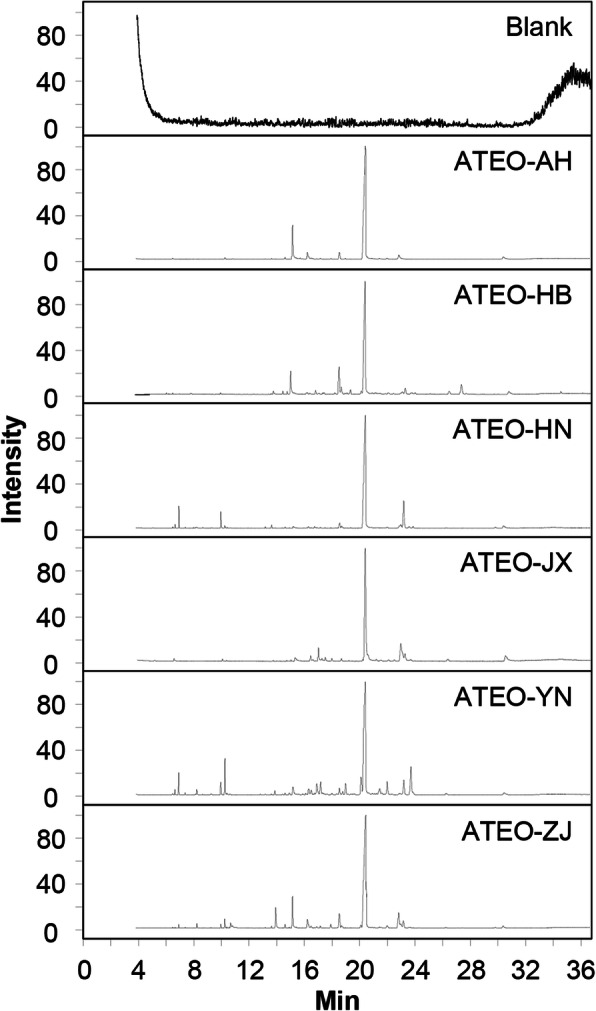
Total ion chromatogram of ATEO. The chromatographic method was described in Methods. The identification of 57 compounds was made by comparing their mass spectra with the mass spectral library (NIST14), with MS spectra and MS fragmentation pattern published in the literature and with Relative Retention Index by injecting a mixture of n-alkanes. Representative chromatograms are shown, *n* = 6

To investigate the variation in chemical composition present in ATEO from six major regions in China, GC-MS analysis was used to detect the holistic volatile component profiles of ATEO. Typical GC-MS spectra of ATEO from six regions are presented in Fig. 1; a wide range of volatile components were unambiguously identified based on mass spectral library (NIST14), MS fragmentation pattern published in the literature, and relative retention index by injecting a mixture of n-alkanes [30–33] (Additional file 2). 57 volatile components, including terpenoids, phenylpropanoids, aromatic compounds, and other aliphatic compounds, were identified and quantification of these components was performed by peak area normalization method, as summarized in Table 2. The primary volatile components, particularly terpenoids, phenylpropanoids and aromatic compounds, were confirmed. The relative contents of terpenoids, phenylpropanoids and aromatic compounds were greatly changed when ATEO from the six major regions were compared.

**Table 2:** Normalized relative content of components from GC/MS of ATEO from six major regions in China

Component	Normalized relative content (%)					
	ATEO-AH	ATEO-HB	ATEO-HN	ATEO-JX	ATEO-YN	ATEO-ZJ
**Terpenoids**						
α-Pinene	–	–	0.82 ± 0.10	–	0.65 ± 0.04	0.14 ± 0.06
Camphene	–	0.21 ± 0.01	4.95 ± 1.10	0.04 ± 0.02	2.63 ± 0.36	0.70 ± 0.34
β-Pinene	–	–	0.23 ± 0.04	–	0.28 ± 0.05	0.17 ± 0.07
α-Terpinene	–	–	0.14 ± 0.02	–	0.04 ± 0.01	–
d-Limonene	–	–	0.25 ± 0.07	–	–	–
Eucalyptol	–	–	0.16 ± 0.00	–	0.70 ± 0.18	0.77 ± 0.19
γ-Terpinene	–	–	0.17 ± 0.03	–	0.07 ± 0.02	0.06 ± 0.01
Linalool	–	–	0.08 ± 0.02	–	0.16 ± 0.08	0.16 ± 0.07
2-Bornanone	0.21 ± 0.12	0.05 ± 0.01	3.45 ± 0.33	0.87 ± 0.24	1.25 ± 0.36	0.62 ± 0.07
endo-Borneol	0.44 ± 0.12	0.30 ± 0.03	0.61 ± 0.07	0.21 ± 0.05	4.02 ± 1.04	1.42 ± 0.15
Terpinen-4-ol	–	–	0.19 ± 0.03	0.06 ± 0.01	0.19 ± 0.03	0.20 ± 0.04
α-Terpineol	0.07 ± 0.02	–	0.06 ± 0.01	0.06 ± 0.01	0.10 ± 0.04	–
δ-Elemene	–	–	–	–	–	0.11 ± 0.05
α-Longipinene	0.10 ± 0.04	–	0.39 ± 0.07	0.04 ± 0.00	0.10 ± 0.03	0.13 ± 0.01
Longicyclene	0.21 ± 0.07	–	0.93 ± 0.14	0.31 ± 0.02	0.18 ± 0.07	0.40 ± 0.02
α-Patchoulene	–	0.18 ± 0.01	–	–	–	–
β-Elemene	0.15 ± 0.04	–	0.16 ± 0.05	0.14 ± 0.03	0.82 ± 0.28	–
Longifolene	0.43 ± 0.29	–	0.11 ± 0.01	0.21 ± 0.19	0.21 ± 0.08	0.06 ± 0.01
β-Caryophyllene	0.78 ± 0.23	0.81 ± 0.04	0.37 ± 0.04	0.30 ± 0.05	0.32 ± 0.01	0.92 ± 0.30
Cedr-8(15)-ene	–	0.14 ± 0.02	0.15 ± 0.07	–	0.24 ± 0.19	0.14 ± 0.07
Calarene	0.20 ± 0.12	0.79 ± 0.10	0.11 ± 0.02	0.48 ± 0.11	0.34 ± 0.02	0.21 ± 0.02
α-Caryophyllene	0.12 ± 0.02	–	–	–	–	0.17 ± 0.07
α-Acoradiene	0.34 ± 0.07	–	–	–	–	–
γ-Muurolene	–	–	–	–	0.30 ± 0.08	0.09 ± 0.04
Germacrene D	–	–	–	–	0.31 ± 0.04	–
Shyobunone	0.43 ± 0.10	1.97 ± 0.21	0.20 ± 0.01	7.96 ± 1.63	2.41 ± 0.75	0.38 ± 0.12
δ-Cadinene	0.34 ± 0.17	0.27 ± 0.06	0.24 ± 0.02	1.17 ± 0.56	3.24 ± 0.57	0.55 ± 0.12
Isoshyobunone	–	1.35 ± 0.13	–	1.91 ± 0.88	4.43 ± 0.82	0.08 ± 0.03
α-Panasinsen	–	0.61 ± 0.08	0.21 ± 0.02	–	–	0.08 ± 0.00
α-Calacorene	–	–	–	1.79 ± 0.95	–	–
Elemol	–	–	–	–	0.51 ± 0.14	–
Eremophila ketone	–	1.98 ± 0.23	0.72 ± 0.18	–	–	0.72 ± 0.16
Germacrene D-4-ol	0.25 ± 0.05	0.24 ± 0.05	–	–	3.25 ± 0.81	0.10 ± 0.02
Spathulenol	–	0.39 ± 0.02	–	0.28 ± 0.10	–	0.09 ± 0.01
Caryophyllene oxide	0.05 ± 0.01	1.66 ± 0.14	0.21 ± 0.02	0.14 ± 0.03	–	0.12 ± 0.01
Viridiflorol	0.05 ± 0.01	–	–	–	0.33 ± 0.12	0.11 ± 0.02
Dehydroxy- isocalamendiol	–	0.64 ± 0.06	–	0.80 ± 0.13	0.14 ± 0.01	0.05 ± 0.01
tau-Cadinol	0.36 ± 0.16	0.38 ± 0.19	–	0.60 ± 0.10	2.64 ± 1.08	0.47 ± 0.18
α-Cadinol	0.36 ± 0.18	0.48 ± 0.18	0.42 ± 0.19	0.82 ± 0.25	4.81 ± 1.23	0.90 ± 0.21
Spiro[4.5]dec-6-en-8-one, 1,7-dimethyl-4- (1-methylethyl)-	0.09 ± 0.03	1.00 ± 0.16	0.83 ± 0.15	0.88 ± 0.18	–	0.52 ± 0.09
Shyobunol	–	–	–	–	9.24 ± 1.64	–
Isocalamenediol	–	1.79 ± 0.56	–	1.92 ± 0.77	0.40 ± 0.07	0.30 ± 0.11
**Phenylpropanoids**						
Methyleugenol	0.11 ± 0.02	0.81 ± 0.08	–	1.11 ± 1.04	–	4.39 ± 0.42
cis-Methyl isoeugenol	14.85 ± 2.08	7.60 ± 0.85	0.85 ± 0.27	3.51 ± 1.41	1.56 ± 0.40	7.46 ± 0.95
Benzene, 1,2-dimethoxy- 4-(1-propenyl)-	3.57 ± 0.88	–	–	–	–	2.46 ± 0.31
Elemicin	0.22 ± 0.07	–	0.12 ± 0.07	–	–	0.86 ± 0.25
γ-Asarone	2.78 ± 0.54	9.39 ± 1.09	2.18 ± 0.76	1.76 ± 0.53	1.28 ± 0.33	3.53 ± 0.67
β-Asarone	69.97 ± 4.24	52.45 ± 4.20	61.38 ± 4.34	55.21 ± 6.48	45.88 ± 7.90	57.62 ± 5.71
α-Asarone	2.78 ± 1.06	0.98 ± 0.29	1.53 ± 0.22	15.12 ± 4.26	0.53 ± 0.20	5.65 ± 1.71
**Aromatic compounds**						
Benzene, (1-methylethyl)-	0.14 ± 0.03	0.22 ± 0.05	0.29 ± 0.04	0.31 ± 0.25	0.16 ± 0.02	0.16 ± 0.03
o-Cymene	–	0.16 ± 0.04	0.16 ± 0.01	–	0.06 ± 0.02	–
Estragole	0.13 ± 0.03	–	–	–	–	1.62 ± 0.17
Aihydroagarofuran	–	–	–	–	0.39 ± 0.07	–
Aristolone	–	8.96 ± 2.95	14.15 ± 2.37	2.90 ± 0.63	5.31 ± 1.04	3.10 ± 1.12
6-Isopropenyl-4,8a-dimethyl-1,2,3,5,6,7,8,8a-octahydronaphthalene-2,3-diol	–	–	0.60 ± 0.20	–	–	0.20 ± 0.04
**Others**						
2-Pentanone, 4-hydroxy-4-methyl-	0.03 ± 0.01	–	–	0.10 ± 0.04	–	0.06 ± 0.01
Bornyl acetate	–	–	0.07 ± 0.02	0.04 ± 0.02	–	0.05 ± 0.01
**Total**	**97.26 ± 2.19**	**92.79 ± 6.71**	**95.22 ± 3.23**	**98.70 ± 4.87**	**94.84 ± 3.38**	**97.72 ± 1.79**

### Significantly changed volatile component analysis in ATEO from six major regions in China

**Fig. 2:**
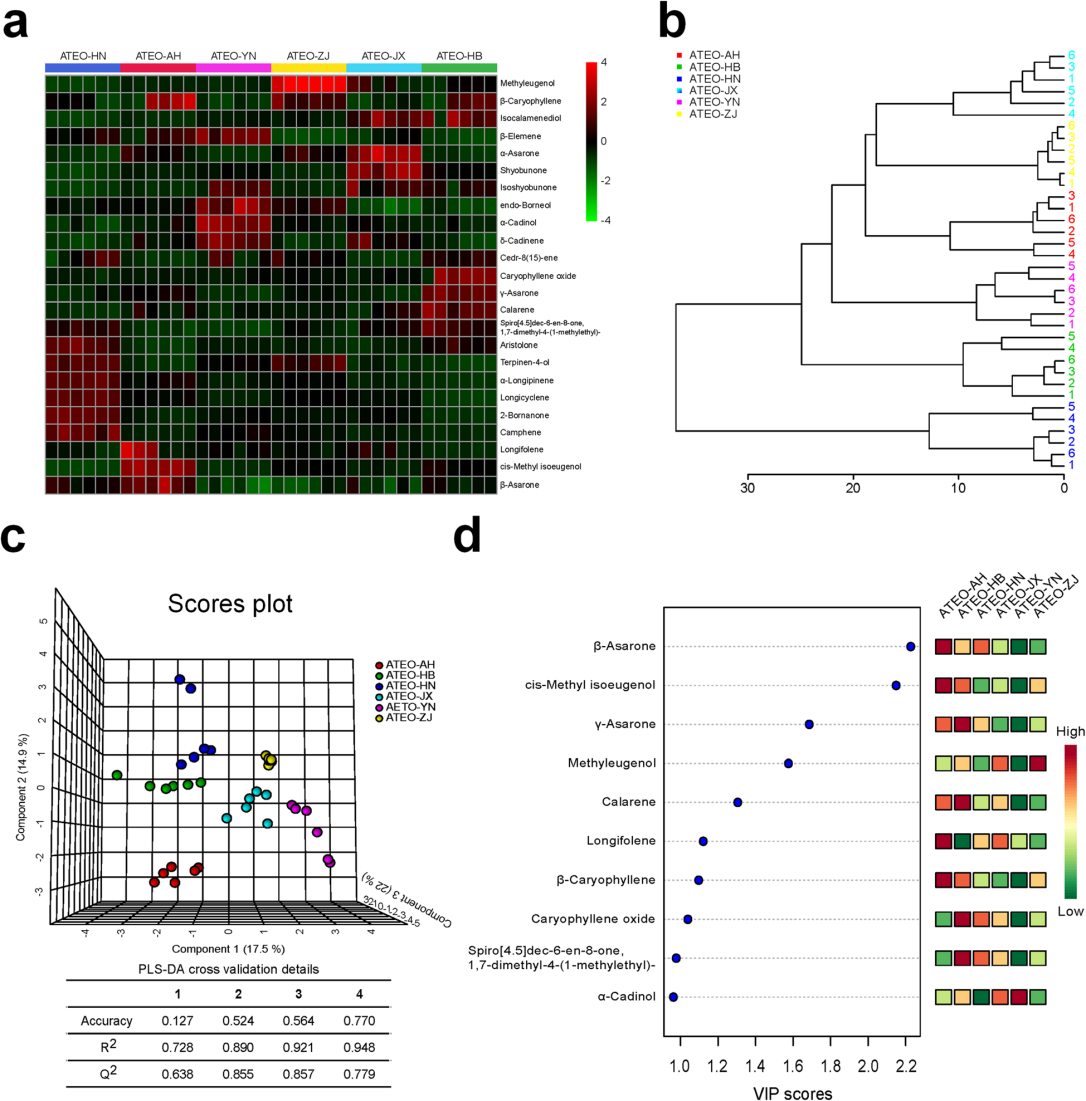
Discriminant analysis of ATEO from six major regions. **a** 24 altered components according to GC-MS visualized by a heat map. **b** PLS-DA model showed that ATEO samples from the six major regions were clearly separated in the X-, Y- and Z-axis direction. Each point represents an independent sample. **c** 10 components with the highest VIP of PC1 were screened out by the PLS-DA model. Top 8 of them with VIP > 1 and *p* < 0.05 were considered as significantly changed volatile components

To comprehensively analyze the information contained in ATEO from six major regions (AH, HB, HN, JX, YN and ZJ), one-way ANOVA, clustering analysis and PLS-DA were applied to classify these data using an online tool, MetaboAnalyst 4.0 (www.metaboanalyst.ca). In one-way ANOVA, 24 components were differentially changed when ATEO from six regions were compared (*p* < 0.05; FDR < 0.05; Table 3), including 18 terpenoids, such as caryophyllene oxide, calarene, cedr-8(15)-ene, spiro[4.5]dec-6-en-8-one, 1,7-dimethyl-4-(1-methylethyl)-, isoshyobunone, isocalamenediol, shyobunol, longifolene, β-caryophyllene, terpinen-4-ol, α-longipinene, longicyclene, 2-bornanone, camphene, α-cadinol, δ-cadinene, β-elemene, and endo-borneol, 5 phenylpropanoids, such as methyleugenol, cis-methyl isoeugenol, γ-asarone, β-asarone, and α-asarone and 1 aromatic compound, such as aristolone. The relative levels of components in ATEO from six regions were visualized on a heat map (Fig. 2a). The clustering analysis was made on 36 ATEO samples from different regions (Fig. 2b). The PLS-DA of the first three components (R^2^X = 0.921 and Q^2^ = 0.857) showed a great separation among ATEO from six regions (Fig. 2c).

**Table 3:** T test of 24 components differentially changed in ATEO samples according to GC-MS analysis

Component	*p*-value	FDR
2-Bornanone	1.06E-14	1.42E-13
Aristolone	1.09E-14	1.42E-13
cis-Methyl isoeugenol	6.02E-14	5.22E-13
Camphene	2.19E-12	1.43E-11
α-Longipinene	7.18E-12	3.39E-11
Longicyclene	7.81E-12	3.39E-11
Terpinen-4-ol	1.35E-11	5.00E-11
α-Asarone	1.58E-10	5.14E-10
γ-Asarone	2.15E-10	6.20E-10
Spiro[4.5]dec-6-en-8-one, 1,7-dimethyl-4-(1-methylethyl)-	1.16E-08	3.02E-08
Calarene	1.56E-07	3.68E-07
Shyobunone	2.10E-07	4.55E-07
α-Cadinol	2.53E-07	4.71E-07
β-Elemene	2.54E-07	4.71E-07
endo-Borneol	3.55E-07	6.15E-07
Methyleugenol	5.14E-07	8.35E-07
β-Asarone	6.19E-07	9.47E-07
Isocalamenediol	1.13E-06	1.63E-06
Caryophyllene oxide	2.16E-06	2.95E-06
δ-Cadinene	7.67E-05	9.98E-05
Cedr-8(15)-ene	0.0027017	0.003345
Isoshyobunone	0.0047441	0.0056067
β-Caryophyllene	0.0079356	0.0089707
Longifolene	0.02099	0.022739

Based on these findings, MCA was used to screen significantly changed volatile components based on the following criteria: VIP > 1 in PLS-DA and *p* < 0.05 in t-tests. 8 volatile components from ATEO were significantly changed due to region diversity (Fig. 2d), as listed in Table 4; these significantly changed volatile components were involved in phenylpropanoids (β-asarone, cis-methyl isoeugenol, γ-asarone and methyleugenol) and terpenoids (calarene, longifolene, β-caryophyllene and caryophyllene oxide). It is worth noting that more than fifteen-fold change in the relative contents of methyleugenol and cis-methyl isoeugenol and less than seven-fold change in the relative contents of β-asarone and γ-asarone, when samples from six regions were compared using this approach.

**Table 4:** Significantly changed volatile components with their formula, compound type and VIP score identified through PLS-DA

Name of components	Formula	Component type	PLS-DA (VIP score)
β-Asarone	C12H16O3	Phenylpropanoids	2.23
cis-Methyl isoeugenol	C11H14O2	Phenylpropanoids	2.15
γ-Asarone	C12H16O3	Phenylpropanoids	1.69
Methyleugenol	C11H14O2	Phenylpropanoids	1.58
Calarene	C15H24	Terpenoids	1.31
Longifolene	C15H24	Terpenoids	1.12
β-Caryophyllene	C15H24	Terpenoids	1.10
Caryophyllene oxide	C15H24O	Terpenoids	1.04

### Variation in the bioactivity of ATEO from six major regions in China

**Fig. 3:**
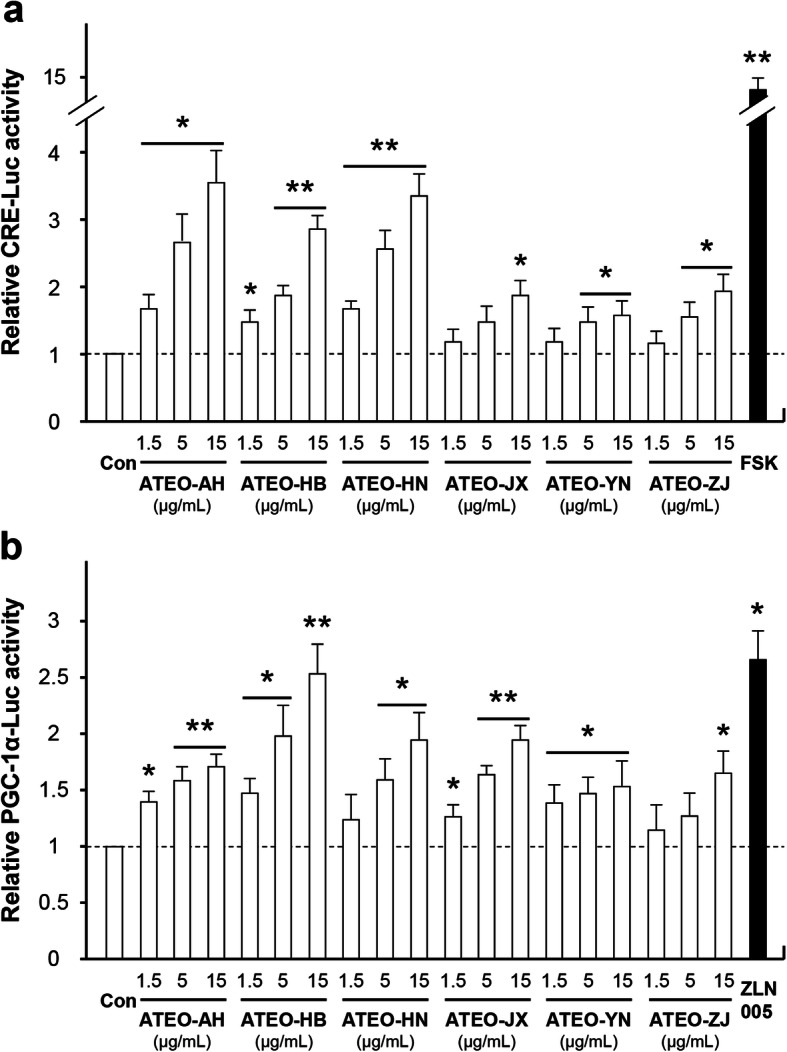
ATEO stimulates transcriptional activities of CRE and PGC-1α in C6 cells. Cultured C6 cells were transfected with pCRE-Luc **a** or pPGC-1α-Luc **b** and subsequently treated with ATEO (1.5, 5, 15 μg/mL) for 24 h. Cells were collected to determine the luciferase activity. Forskolin (FSK, 10 μM) and ZLN005 (2 μM) were used as positive control. Data are expressed as fold of control (untreated culture), and in mean ± SEM, where *n* = 3. *p < 0.05, **p < 0.01 compared with control

The bioactivity of ATEO from six major regions in China was screened by C6 glioma cells. A cell viability assay was performed to determine a safe concentration range (0–15 μg/mL) of ATEO (the concentrations at which all extracts did not induce cell proliferation or death; Additional file 3). The inflammation response could be triggered by co-treatment with TNF-α and IFN-γ in cultured C6 cells [25]. Treatment of TNF-α and IFN-γ (10 + 10 ng/mL) showed the maximal induction of interleukin-1β (IL-1β), interleukin-6 (IL-6) and TNF-α mRNA expression in cultured C6 cells and this concentration was selected for further study (Additional file 4). cAMP-response element binding protein (CREB) and PGC-1α are master regulators of anti-inflammation, anti-oxidation and neurotrophic factor expression during neuroprotection. The up-regulation of CREB and PGC-1α expression induces the expression of several anti-oxidant and anti-inflammatory proteins as well as neurotrophic factors [34–36]. Therefore, the transcriptional activities of CRE and PGC-1α were selected for dosage screening of ATEO. ATEO samples induced CRE and PGC-1α transcriptional activities in dose-dependent manners: the maximal induction of promoter activities was observed at ~ 15 μg/mL (Fig. 3).

**Fig. 4:**
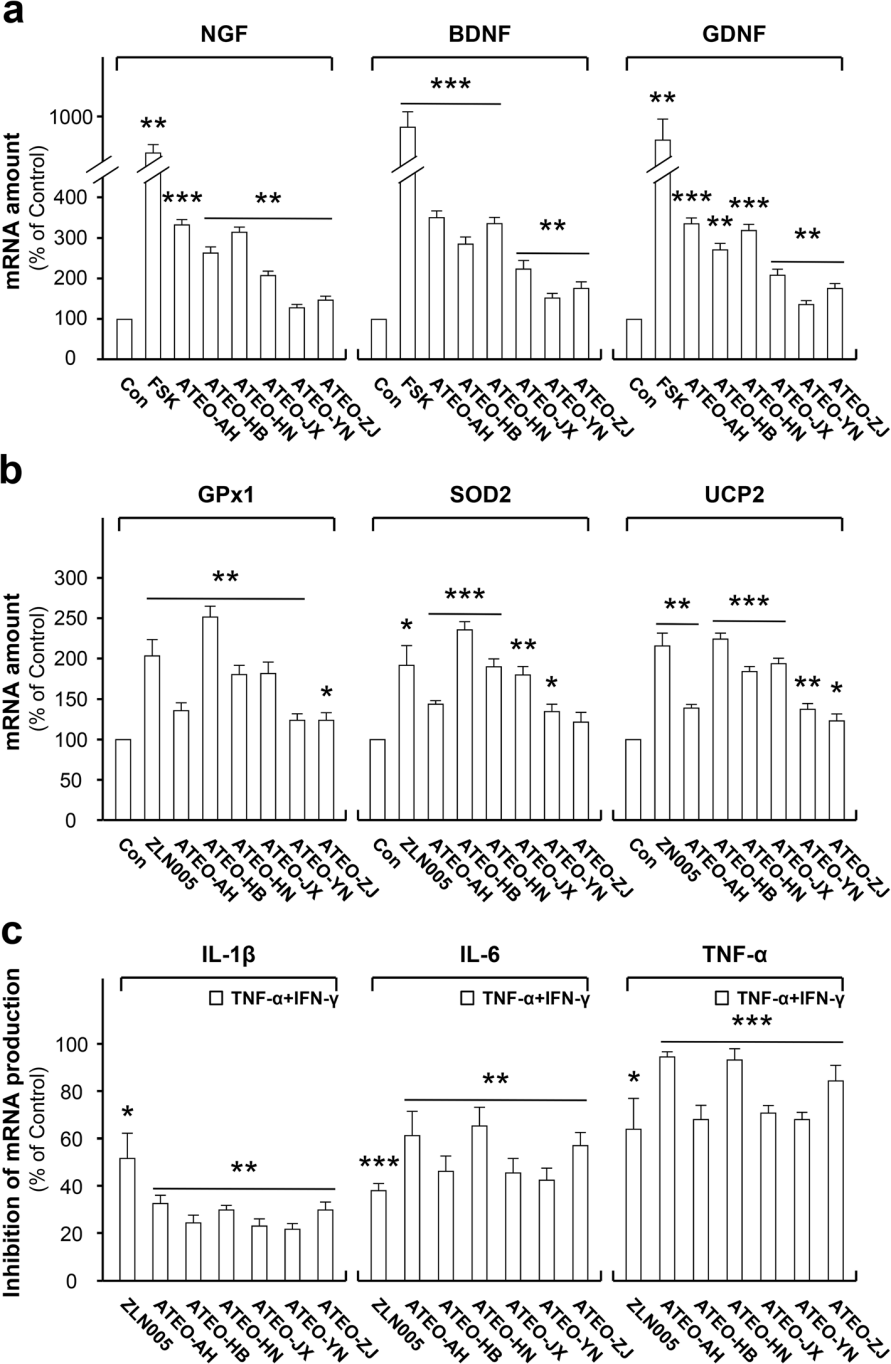
ATEO regulates the expression of neuroprotective proteins in C6 cells. In neurotrophic (NGF, BDNF and GDNF, **a**) and anti-oxidation (GPx1, SOD2 and UCP2, **b**) activity, cultured C6 cells were treated with ATEO (15 μg/mL) for 48 h. In anti-inflammation (IL-1β, IL-6 and TNF-α, **c**) activity, cells were treated with ATEO (15 μg/mL) for 48 h before TNF-α and IFN-γ (10 + 10 ng/mL) co-treatment for another 24 h. Cells were collected to determine the mRNA amount. Forskolin (FSK, 10 μM) and ZLN005 (2 μM) were used as positive control. Data are expressed as percentage of control (untreated culture), and in mean ± SEM, where *n* = 3. *p < 0.05, **p < 0.01, ***p < 0.001 compared with control

**Fig. 5:**
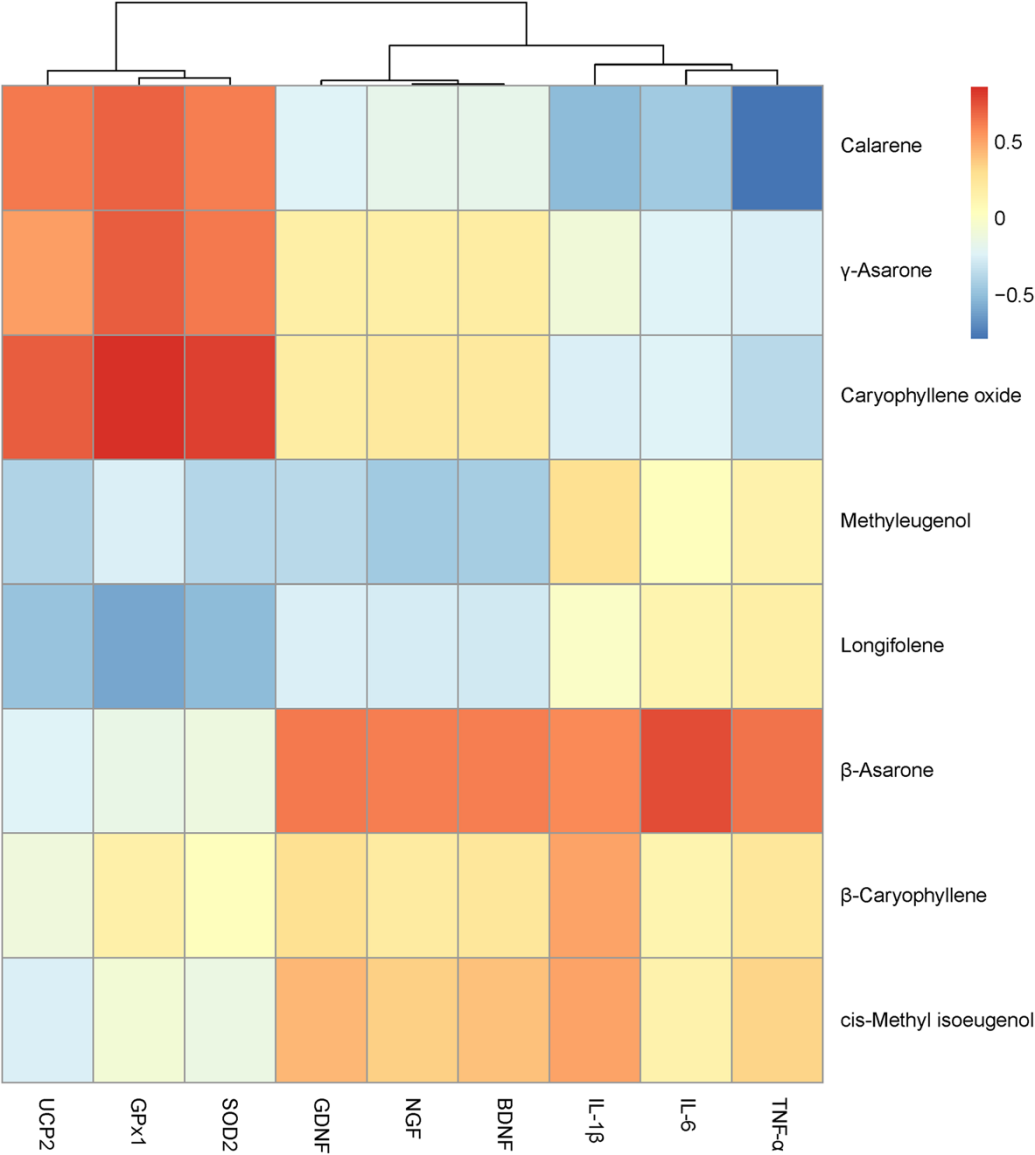
Correlation analysis between GC/MS data and neuroprotective activity. Correlation analysis was performed for the activity data of neurotrophic factors (NGF, BDNF and GDNF), anti-oxidation (GPx1, SOD2 and UCP2) and anti-inflammation (IL-1β, IL-6 and TNF-α) and relative contents of significantly changed volatile components in ATEO using RStudio 1.1.463. The results were visualized on a heat map

The bioactivities of anti-inflammation (IL-1β, IL-6 and TNF-α), anti-oxidation [glutathione peroxidase 1 (GPx1), superoxide dismutase 2 (SOD2) and uncoupling protein2 (UCP2)] and neurotrophic [nerve growth factor (NGF), brain-derived neurotrophic factor (BDNF) and glial cell-derived neurotrophic factor (GDNF)] were selected for further study. The results indicated that the ATEO samples (15 μg/mL) significantly inhibited inflammation by reducing the expression of pro-inflammatory cytokines (IL-1β, IL-6 and TNF-α), and had great anti-oxidant and neurotrophic effects in promoting the expression of anti-oxidant proteins (GPx1, SOD2 and UCP2) and neurotrophic factors (NGF, BDNF and GDNF) (Fig. 4). Next, we performed correlation analysis between the bioactivities (IL-1β, IL-6, TNF-α, GPx1, SOD2, UCP2, NGF, BDNF and GDNF) and the significantly changed volatile components using RStudio 1.1.463 (www.rstudio.com/) (Fig. 5). The correlation coefficients between the bioactivities and the components were shown in Table 5. The correlation ranges |r| ≥ 0.6 and *p* < 0.05 were considered as strong correlation [37]. The anti-inflammatory and neurotrophic activity of ATEO was strongly and positively correlated with the content of β-asarone. The anti-oxidant activity of ATEO was strongly and positively correlated with the content of γ-asarone. The results indicated strong correlation between the bioactivities and significantly changed volatile components (β-asarone and γ-asarone). Therefore, these significantly changed volatile components could be served as volatile active components in ATEO which reflect the quality variation of ATEO in response to region difference. The results might be useful for the development of quality study of ATEO.

**Table 5:** Correlation coefficients between bioactivities and relative contents of significantly changed volatile components in ATEO

Component	NGF	BDNF	GDNF	GPx1	SOD2	UCP2	IL-1β	IL-6	TNF-α
β-Asarone	0.618	0.621	0.636	− 0.142	− 0.106	− 0.220	0.600	0.772	0.659
cis-Methyl isoeugenol	0.366	0.407	0.440	−0.066	− 0.125	− 0.237	0.505	0.151	0.351
γ-Asarone	0.188	0.196	0.182	0.724	0.643	0.524	−0.078	−0.223	− 0.249
Methyleugenol	−0.439	−0.431	− 0.364	−0.251	− 0.366	−0.396	0.303	0.056	0.149
Calarene	−0.154	−0.167	− 0.227	0.709	0.632	0.647	−0.506	−0.435	− 0.776
Longifolene	−0.253	− 0.276	−0.249	− 0.595	−0.503	− 0.466	−0.002	0.136	0.175
β-Caryophyllene	0.223	0.244	0.294	0.168	0.059	−0.095	0.508	0.132	0.254
Caryophyllene oxide	0.233	0.224	0.191	0.862	0.810	0.722	−0.246	−0.226	−0.364

## Discussion

The quality study of Chinese herbal medicine is a scientific problem and an industrial issue which hampers the development of Chinese medicine [38]. Region difference plays a key role in heterogeneous quality of Chinese herbal medicine and final drugs as the chemical composition and biological activity of the herbs vary with region [3, 39]. However, current quality control approaches for Chinese herbal medicine are mostly focused on some targeted components which lack overall evaluation of the impact of region. Thus, the impact of region should be taken seriously and finding ways to explore the quality study of Chinese herbal medicine is of great importance.

ATR is one of the most important Chinese herbal medicines in treating neurological disorders. The primary active fraction of ATR is the essential oil. ATEO was reported to exert a neuroprotective effect in neurological disorders, including in neuroprotection on anti-oxidation, anti-inflammation and neurotrophic function [12–15]. The quality of ATEO varies widely due to region difference; however, little is known about how to study ATEO quality chemically and biologically in response to region difference. In this work, we identified volatile active components in ATEO from six major regions in China using chemical component analysis combined with biological activity evaluation to explore the quality study with region difference.

In the study, six major regions of ATR were involved, i.e. mountain areas in AH, HB and YN, valley area in HN, hilly area in JX and hilly basin area in ZJ province. Some details of the geographical environment in six major regions were shown (Additional file 1). The variation in geographical environment resulted in diversity of secondary metabolites. Amongst all the secondary metabolites, β-asarone is considered as the major active component (45.88–69.97% relative content), the content of which showed significant differences between groups (AH and HB; AH and JX; AH and YN; AH and ZJ; HB and YN; HB and ZJ; HN and JX; HN and YN; HN and ZJ). It is interesting that ATEO-AH, ATEO-HB and ATEO-YN are from the mountain areas but ATEO from YN province in southwest China had lower content of β-asarone than that of samples in southeast China. The synthesis of β-asarone is promoted by phenylalanine ammonia lyase (PAL), one of the key enzymes in phenylpropanes metabolism pathway [40]. The activity of PAL is affected by several exogenous factors, such as light, temperature, growth regulators, pathogen infection, injury, etc. [41]. It is reported that YN is the major mineral-producing province in China, and a lot of slag, waste gas and sewage are produced during the mining of minerals, causing soil heavy metal pollution. Heavy metal with high levels in soil causes toxic effects and decreases PAL activity [42], which may explain the low content of β-asarone in YN samples. From this point of view, the southeast regions might be suitable for ATR harvesting. Furthermore, much more effort is required to elucidate the reasons for the variation in chemical composition of ATEO samples.

The bioactivity assays studied in this work included regulation of anti-oxidative proteins (GPx1, SOD2 and UCP2), neurotrophic factors (NGF, BDNF and GDNF) and pro-inflammatory cytokines (IL-1β, IL-6 and TNF-α). The up-regulation of anti-oxidative proteins (GPx1, SOD2 and UCP2) and inhibition of pro-inflammatory cytokines (IL-1β, IL-6 and TNF-α) could protect neurons against damage caused by injury, oxidative stress and inflammation, while the induction of neurotrophic factors could promote neuroregeneration [14, 43]. Our results indicated that ATEO exhibited anti-oxidative, anti-inflammatory and neurotrophic effects which were supported by previous findings that ATEO protected H_2_O_2_-induced cell injury via CREB/PGC-1α activation in PC12 cells and induced the expression of neurotrophic factors by protein kinase A (PKA) in cultured astrocytes [15, 44]. The ATEO mediated-regulation of anti-oxidative effect was strongly correlated with the level of γ-asarone, while the regulation of anti-inflammatory and neurotrophic activity by ATEO was closely related to the level of β-asarone. These activities of ATEO may be relevant to the traditional use of ATR: re-gaining consciousness, tranquilizing the mind, eliminating dampness, and invigorating the circulation of blood [45].

Based on these findings, 2 components (β-asarone and γ-asarone) could be served as volatile active components of ATEO in response to region difference. β-Asarone (cis double bond) and α-asarone (tran double bond) are sis-trans isomer in ATEO. They exert various neuroprotective functions, such as anti-oxidation, promoting neuronal differentiation and neurotrophic factor expression [13, 15, 46]. It is worth noting that β-asarone showed greater effects on promoting neuronal differentiation and BDNF expression than that of α-asarone, while these two asarones presented similar effect on anti-oxidation. These results indicate that double bond isomerization might play an important role in neurotrophic function, while the conversion of cis and trans isomers did not affect anti-oxidant property. Furthermore, much more effort is required to elucidate the structure and biological activities of asarones in ATEO.

## Conclusions

In this study, the volatile active components of ATEO from six major regions in China were investigated by using an astrocyte-like cell line, C6 glioma cells. It was indicated that 8 components (β-asarone, cis-methyl isoeugenol, γ-asarone, methyleugenol, terpenoids, calarene, longifolene, β-caryophyllene and caryophyllene oxide) in ATEO were significantly changed in response to region difference. And 2 components (β-asarone and γ-asarone) showed strong correlation with the biological activities (anti-oxidative, anti-inflammatory and neurotrophic effects) which could be served as the volatile active components in quality study. These findings provide an insight into ATEO quality, which will be useful for the development of quality study of ATEO; further research should focus on validating the volatile active components of ATEO in cell and animal models.

## Supplementary information

**Additional file 1.** Details of geographical environment of six major regions.

**Additional file 2.** Identification of components in ATEO.

**Additional file 3.** Effect of ATEO on the growth of cultured C6 cells.

**Additional file 4.** Effect of TNF-α and IFN-γ on the pro-inflammatory cytokine mRNA expression of cultured C6 cells.

**Additional file 5.** The original rPCR data of ATEO bioactivity.

## Data Availability

All data generated or analyzed during this study are included in this published article and its supplementary information files.

## Abbreviations

ATR: Acori Tatarinowii Rhizome
ATEO: Acori Tatarinowii Rhizome essential oil
GC-MS: Gas Chromatography-Mass Spectrometer
DAL: Drug Administration Law
GAP: Good Agricultural Practices
AH: Anhui
HB: Hubei
HN: Hunan
JX: Jiangxi
YN: Yunnan
ZJ: Zhejiang
PLS-DA: partial least-squares discriminant analysis
ANOVA: Analysis of Variance
EI: Electron impact
ATCC: American Type Culture Collection
DMEM: Dulbecco’s modified Eagle’s medium
FBS: Fetal bovine serum
FSK: Forskolin
TNF-α: Tumor necrosis factor-α
IFN-γ: Interferon-γ
CRE: cAMP response element
PGC-1α: Peroxisome proliferator-activated receptor-γ coactivator-1α
MCA: Multi-criteria assessment
VIP: Variable importance in projection
FDR: False discovery rate
IL-1β: Interleukin-1β
IL-6: Interleukin-6
CREB: cAMP-response element binding protein
GPx1: Glutathione peroxidase 1
SOD2: Superoxide Dismutase 2
UCP2: Uncoupling protein2
NGF: Nerve growth factor
BDNF: Brain-derived neurotrophic factor
GDNF: Glial cell-derived neurotrophic factor
PAL: Phenylalanine ammonia lyase
PKA: Protein kinase A
