# Supplementary figures and images for: Identification of volatile active components in Acori Tatarinowii Rhizome essential oil from different regions in China by C6 glioma cells
Source: BMC Complement Med Ther. 2020 Aug 17;20:255. doi: 10.1186/s12906-020-03020-4 (PMC7430108; doi:10.1186/s12906-020-03020-4)

**Additional file 1.** Details of geographical environment of six major regions.


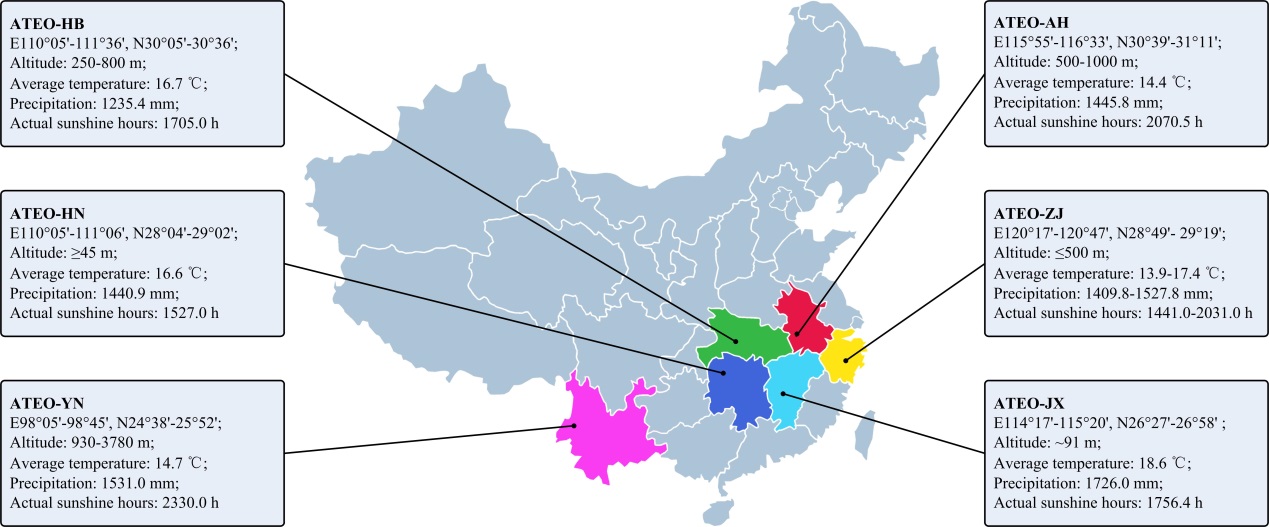

Supplement: Supplementary file 1 — Additional file 1. Details of geographical environment of six major regions. [file 12906_2020_3020_MOESM1_ESM.docx]
