# Supplementary material for: Identification of volatile active components in Acori Tatarinowii Rhizome essential oil from different regions in China by C6 glioma cells
Source: BMC Complement Med Ther. 2020 Aug 17;20:255. doi: 10.1186/s12906-020-03020-4 (PMC7430108; doi:10.1186/s12906-020-03020-4)
Supplement: Supplementary file 2 — Additional file 2. Identification of components in ATEO. [file 12906_2020_3020_MOESM2_ESM.docx]

**Additional file 2.** Identification of components in ATEO.

| No. | Retention Time (min) | KI | Component name | Formula | Molecular Weight | References |
| --- | --- | --- | --- | --- | --- | --- |
| 1 | 5.22 | 844.51 | 2-Pentanone, 4-hydroxy-4-methyl- | C6H12O2 | 116 | Database |
| 2 | 6.62 | 930.49 | Benzene, (1-methylethyl)- | C9H12 | 120 | Database |
| 3 | 6.78 | 940.24 | α-Pinene | C10H16 | 136 | Database |
| 4 | 7.05 | 956.10 | Camphene | C10H16 | 136 | Database |
| 5 | 7.50 | 984.15 | β-Pinene | C10H16 | 136 | Database |
| 6 | 8.10 | 1023.49 | α-Terpinene | C10H16 | 136 | Database |
| 7 | 8.25 | 1031.54 | o-Cymene | C10H14 | 134 | Database |
| 8 | 8.30 | 1036.24 | d-Limonene | C10H16 | 136 | Database |
| 9 | 8.35 | 1038.93 | Eucalyptol | C10H18O | 154 | Database |
| 10 | 8.77 | 1065.77 | γ-Terpinene | C10H16 | 136 | Database |
| 11 | 9.38 | 1106.67 | Linalool | C10H18O | 154 | Database |
| 12 | 10.04 | 1157.78 | 2-Bornanone | C10H16O | 152 | Database |
| 13 | 10.33 | 1179.26 | endo-Borneol | C10H18O | 154 | Database |
| 14 | 10.49 | 1189.63 | Terpinen-4-ol | C10H18O | 154 | Database |
| 15 | 10.66 | 1207.04 | α-Terpineol | C10H18O | 154 | Database |
| 16 | 10.74 | 1208.45 | Estragole | C10H12O | 148 | Database |
| 17 | 11.88 | 1291.43 | Bornyl acetate | C12H20O2 | 196 | Database |
| 18 | 12.84 | 1346.07 | δ-Elemene | C15H24 | 204 | Database |
| 19 | 13.20 | 1366.29 | α-Longipinene | C15H24 | 204 | Database |
| 20 | 13.64 | 1391.01 | Longicyclene | C15H24 | 204 | Database |
| 21 | 13.82 | 1400.85 | α-Patchoulene | C15H24 | 204 | Database |
| 22 | 13.89 | 1403.83 | β-Elemene | C15H24 | 204 | Database |
| 23 | 13.99 | 1407.23 | Methyleugenol | C11H14O2 | 178 | Database |
| 24 | 14.37 | 1424.26 | Longifolene | C15H24 | 204 | Database |
| 25 | 14.61 | 1434.04 | β-Caryophyllene | C15H24 | 204 | Database |
| 26 | 14.72 | 1440.00 | Cedr-8(15)-ene | C15H24 | 204 | Database |
| 27 | 14.91 | 1446.81 | Calarene | C15H24 | 204 | Database |
| 28 | 15.13 | 1456.60 | cis-Methyl isoeugenol | C11H14O2 | 178 | Database |
| 29 | 15.40 | 1467.66 | α-Caryophyllene | C15H24 | 204 | Database |
| 30 | 15.69 | 1480.43 | α-Acoradiene | C15H24 | 204 | Database |
| 31 | 15.86 | 1487.66 | γ-Muurolene | C15H24 | 204 | Database |
| 32 | 16.06 | 1496.14 | Germacrene D | C15H24 | 204 | Database |
| 33 | 16.20 | 1501.58 | Benzene, 1,2-dimethoxy-4-1-propenyl- | C11H14O2 | 178 | Database |
| 34 | 16.70 | 1517.41 | Aihydroagarofuran | C15H26O | 222 | Database |
| 35 | 16.87 | 1522.78 | Shyobunone | C15H24O | 220 | Database |
| 36 | 17.12 | 1530.70 | δ-Cadinene | C15H24 | 204 | Database |
| 37 | 17.34 | 1537.66 | Isoshyobunone | C15H24O | 220 | Database |
| 38 | 17.43 | 1540.82 | α-Panasinsen | C15H24 | 204 | Liu *et al.*^[29]^; Database |
| 39 | 17.81 | 1552.85 | α-Calacorene | C15H20 | 200 | Database |
| 40 | 17.86 | 1553.80 | Elemicine | C12H16O3 | 208 | Database |
| 41 | 17.92 | 1563.92 | Elemol | C15H26O | 222 | Database |
| 42 | 18.47 | 1573.10 | γ-Asarone | C12H16O3 | 208 | Satyal *et al.*^[30]^; Database |
| 43 | 18.62 | 1578.48 | Eremophila ketone | C15H24O | 220 | Database |
| 44 | 18.89 | 1586.71 | Germacrene D-4-ol | C15H26O | 222 | Database |
| 45 | 19.01 | 1590.51 | Spathulenol | C15H24O | 220 | Database |
| 46 | 19.23 | 1597.78 | Caryophyllene oxide | C15H24O | 220 | Database |
| 47 | 19.55 | 1606.52 | Viridiflorol | C15H26O | 222 | Database |
| 48 | 20.30 | 1622.95 | β-Asarone | C12H16O3 | 208 | Database |
| 49 | 20.97 | 1640.10 | Dehydroxy-isocalamendiol | C15H24O | 220 | Database |
| 50 | 21.29 | 1648.55 | tau-Cadinol | C15H26O | 222 | Database |
| 51 | 21.84 | 1661.11 | α-Cadinol | C15H26O | 222 | Database |
| 52 | 23.04 | 1681.88 | α-Asarone | C12H16O3 | 208 | Database |
| 53 | 23.41 | 1700.97 | Spiro[4.5]dec-6-en-8-one,1,7-dimethyl-4-(1-methylethyl)- | C15H24O | 220 | Dong *et al.*^[31]^; Database |
| 54 | 23.54 | 1701.74 | Shyobunol | C15H26O | 222 | Dave *et al.*^[32]^; Database |
| 55 | 26.03 | 1749.81 | Isocalamenediol | C15H26O2 | 238 | Database |
| 56 | 26.87 | 1766.02 | Aristolone | C15H22O | 218 | Database |
| 57 | 29.56 | 1825.34 | 6-Isopropenyl-4,8a-dimethyl-1,2,3,5,6,7,8,8a-octahydronap hthalene-2,3-diol | C15H24O2 | 236 | Database |
