# Supplementary material for: Identification of volatile active components in Acori Tatarinowii Rhizome essential oil from different regions in China by C6 glioma cells
Source: BMC Complement Med Ther. 2020 Aug 17;20:255. doi: 10.1186/s12906-020-03020-4 (PMC7430108; doi:10.1186/s12906-020-03020-4)
Supplement: Supplementary file 3 — Additional file 3. Effect of ATEO on the growth of cultured C6 cells. [file 12906_2020_3020_MOESM3_ESM.docx]

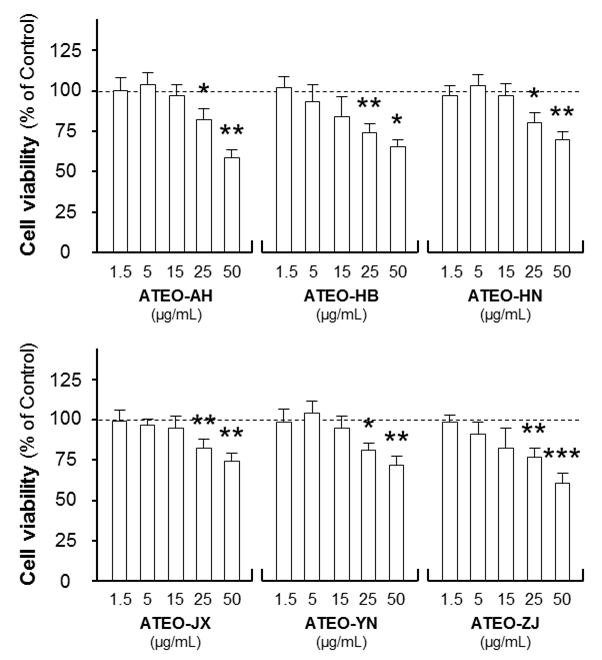


**Additional file 3:** Effect of ATEO on the growth of cultured C6 cells.

Cultured C6 cells were treated with ATEO (0-50 μg/mL) for 48 hours. A cell viability and proliferation test (using the colorimetric MTT assay) was performed. Data are expressed as mean ± SEM, where *n* = 5. **p* < 0.05; ***p* < 0.01; ****p* < 0.001 compared with control.
