# Supplementary material for: Identification of volatile active components in Acori Tatarinowii Rhizome essential oil from different regions in China by C6 glioma cells
Source: BMC Complement Med Ther. 2020 Aug 17;20:255. doi: 10.1186/s12906-020-03020-4 (PMC7430108; doi:10.1186/s12906-020-03020-4)
Supplement: Supplementary file 4 — Additional file 4. Effect of TNF-α and IFN-γ on the pro-inflammatory cytokine mRNA expression of cultured C6 cells. [file 12906_2020_3020_MOESM4_ESM.docx]

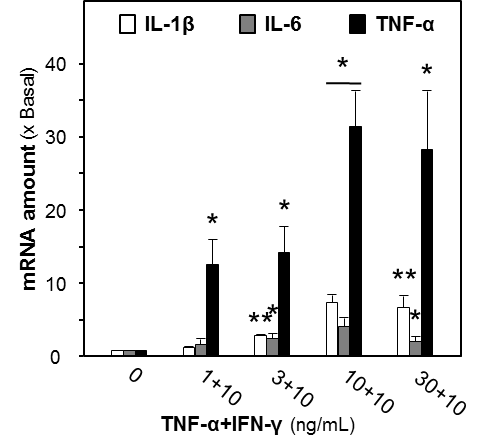


**Additional file 4:** Effect of TNF-α and IFN-γ on the pro-inflammatory cytokine mRNA expression of cultured C6 cells.

Cultured C6 cells were treated with TNF-α and IFN-γ (1+10, 3+10, 10+10, 30+10 ng/mL) for 24 h. Cells were collected to determine the mRNA amount. Data are expressed as fold of control (untreated culture), and in mean ± SEM, where *n* = 3. **p* < 0.05; ***p* < 0.01 compared with control.
